# Targeting PTBP1 blocks glutamine metabolism to improve the cisplatin sensitivity of hepatocarcinoma cells through modulating the mRNA stability of glutaminase

**DOI:** 10.1515/med-2023-0756

**Published:** 2023-09-12

**Authors:** Ruimin Tian, Yanfei Li, Xiaojie Shen, Ying Li

**Affiliations:** Liver Diseases Branch, Tianjin Second People’s Hospital, Tianjin, 300192, China; Department of Infectious, People’s Hospital of Huan County, Qingyang, Gansu, 745700, China; Department of Infectious, Tianjin Second People’s Hospital, No. 7 Sudi South Road, Nankai District, Tianjin, 300192, China

**Keywords:** hepatocellular carcinoma, PTBP1, RNA-binding protein, glutamine metabolism, glutaminase, chemosensitivity, CDDP resistance

## Abstract

Hepatocellular carcinoma (HCC) is a frequently diagnosed malignancy with a high mortality rate. Cisplatin (CDDP) is a widely applied anti-cancer drug. However, a large population of liver cancer patients developed CDDP resistance. The polypyrimidine tract binding protein (PTBP1) is an RNA-binding protein involving in progressions of diverse cancers. Here we report PTBP1 was significantly upregulated in liver tumors and cell lines. Silencing PTBP1 effectively sensitized HCC cells to CDDP. From the established CDDP-resistant HCC cell line (HepG2 CDDP Res), we observed that CDDP-resistant cells were more sensitive to CDDP under low glutamine supply compared with that in HCC parental cells. CDDP-resistant HCC cells displayed elevated glutamine metabolism rate. Consistently, PTBP1 promotes glutamine uptake and the glutamine metabolism key enzyme, glutaminase (GLS) expression. Bioinformatics analysis predicted that the 3′-UTR of GLS mRNA contained PTBP1 binding motifs which were further validated by RNA immunoprecipitation and RNA pull-down assays. PTBP1 associated with GLS 3′-UTR to stabilize GLS mRNA in HCC cells. Finally, we demonstrated that the PTBP1-promoted CDDP resistance of HCC cells was through modulating the GLS–glutamine metabolism axis. Summarily, our findings uncovered a PTBP1-mediated CDDP resistance pathway in HCC, suggesting that PTBP1 is a promisingly therapeutic target to overcome chemoresistance of HCC.

## 1 Introduction

Liver cancer, which is a common malignancy with a high mortality rate and poor prognosis, is one of the most leading causes of cancer-associated death [1,2]. Hepatocellular carcinoma (HCC) is the major (70–85%) subtype of liver cancer [3]. Currently, surgical removal is the primarily therapeutic approach against HCC [4]. However, early symptoms of primary HCC are difficult to characterize [4]. Thus, chemo- and radio-therapies have been applied to HCC patients in middle or advanced stage when significant symptoms were observed [5]. Cisplatin (CDDP) is a platinum-based chemotherapeutic drug, acting through interacting with purine bases to induce DNA lesions, leading to cancer cell death [5,6]. Although CDDP has been widely used for cancer treatment in clinical practice, side effects and development of CDDP resistance has resulted in substantial barriers to limit its wide applications [7]. Therefore, it is imperative to identify the molecular pathways and mechanisms of CDDP-resistant HCC.

Tumor cells exhibit metabolic profiles that they demand higher metabolic rates such as glucose and glutamine metabolism [8]. Accumulating studies uncovered critical roles of glutamine metabolism in tumorigenesis and development using *in vitro* and *in vivo* models [9,10]. Moreover, the dysregulated cellular metabolism rate of cancer cells was tightly correlated to chemoresistance [11] suggesting that blocking glutamine metabolism of HCC cells could potentially enhance the therapeutic outcomes of CDDP.

Polypyrimidine tract binding protein (PTBP1) is an RNA-binding protein which plays important biological roles in diverse processes of cancer cells [12]. PTBP1 was known to act as a tumor-promotive regulator in various cancers, including gastric cancer [13], breast cancer [14], colon cancer [15], and lung cancer [16]. Furthermore, studies have proven that PTBP1 was positively associated with glucose metabolism of cancer cells through modulating the pyruvate kinase M2 isoform [17]. However, the precise roles and molecular targets of PTBP1 in regulating glutamine metabolism and CDDP resistance of liver cancer cells have not been elucidated.

In this study, the biological roles of PTBP1 in CDDP-resistant liver cancer were investigated. Bioinformatics analysis predicted the 3′-UTR of glutaminase (GLS) contained binding motifs of PTBP1. Molecular mechanisms of the PTBP1–GLS association were explored. This study proposes that targeting the PTBP1–GLS–glutamine metabolism axis could be a potential strategy to overcome chemoresistance of liver cancer.

## 2 Materials and methods

### 2.1 HCC specimen collections

This study was approved by the Ethics Committee of Tianjin Second People’s Hospital. Forty HCC tumor specimens and matched normal liver tissues were collected from liver cancer patients from July 2018 to April 2020 in the Liver Diseases Branch, Tianjin Second People’s Hospital, Tianjin, China. No chemo- or radio-therapy was applied before biopsy. After surgery, tissues were immediately stored in liquid nitrogen. All patients signed the informed consent. The required minimum sample number (*n* = 35) was estimated by power analysis.

### 2.2 Cell culture and reagents

HCC cell lines HepG2, Huh7, CA3, SNU-878, and SNU-182 as well as human normal liver cell line, L02 were purchased from the Shanghai Institute for Biological Science (China). Cells were cultured with RPMI 1640 medium with 10% fetal bovine serum plus 100 U/mL penicillin and 100 U/mL streptomycin under 5% CO_2_ at 37°C. Establishment of CDDP-resistant HepG2 cell line was performed according to previous descriptions [18]. Monoclonal anti-PTBP1 (rabbit, #8776), anti-GLS (rabbit, #56750), anti-MDR1/ABCB1 (rabbit, #13342), and anti-β-actin (rabbit, #4970) were purchased from Cellsignaling Tech (Danvers, MA, USA). CDDP was purchased from Sigma-Aldrich (Shanghai, China).

### 2.3 Bioinformatics analysis

The association between PTBP1 and GLS and the binding motifs of PTBP1 on 3′-UTR of GLS were predicted from starBase of ENCORI http://starbase.sysu.edu.cn/. Kaplan–Meier plotter survival rate for liver cancer patients with low or high PTBP1 level was analyzed from https://kmplot.com. Expressions of PTBP1 and GLS from normal or tumorous liver tissues were analyzed from TCGA database by https://ualcan.path.uab.edu. The expression correlation between PTBP1 and GLS in liver cancer was analyzed by Pearson correlation coefficient analysis from starBase of ENCORI http://starbase.sysu.edu.cn/.

### 2.4 Transfection with siRNA or plasmid DNA

Silencing of GLS or PTBP1 gene expression was conducted by transfection of specific siRNA. HepG2 cells were incubated in six-well plates at a density of 5 × 10^5^ cells/well for overnight. Cells were transfected with siRNA targeting human GLS or PTBP1 using the Lipofectamine 3000 reagent (Invitrogen, Carlsbad, CA, USA) according to the manufacturer’s instructions. PTBP1 overexpression plasmid and control plasmid were obtained from Origene.com. The control siRNA, siGLS, and siPTBP1 were synthesized by Gene-Pharma Co. (Shanghai, China). siRNA knockdown efficiency was validated by western blot in HCC cells. Plasmid DNA was transfected at 1 µg/mL and siRNAs were transfected at 25 nM for 48 h. Transfection was performed in triplicate.

### 2.5 Total RNA isolation and qRT-PCR

Total RNA was isolated using the TRIzol reagent (Invitrogen, Carlsbad, CA, USA) according to the manufacturer’s instructions. Briefly, cultured cells were lysed with lysis buffer and proteinase K. DNase I solution (Thermo Fisher Scientific) was added to digest the genomic DNA. The quality and quantity of RNA samples were measured by a NanoDrop ND-2000 spectrophotometer (Thermo Scientific, Rockford, IL, USA). The first-strand cDNA was synthesized from RNA sample (1 µg) using iScript™ RT Supermix for RT-qPCR® (Bio-Rad, Hercules, CA, USA). The qRT-PCR reactions were conducted using the SYBR Green qPCR SuperMix reagents (Thermo Fisher Scientific Inc., Waltham, MA, USA) with typical amplification parameters of 95°C for 30 s, followed by 40 cycles at 98°C for 10 s and 60°C for 30 s. Primers for qRT-PCR were: PTBP1: Forward: 5′-GCATCGACTTTTCCAAGCTC-3′, Reverse: 5′-GGAAACCAGCTCCTGCATAC-3′; GLS: Forward: 5′-CAGAAGGCACAGACATGGTTGG-3′, Reverse: 5′-GGCAGAAACCACCATTAGCCAG-3′; β-actin: Forward: 5′-CTGAGAGGGAAATCGTGCGT-3′, Reverse: 5′-CCACAGGATTCCATACCCAAGA-3′. β-Actin was used as an internal control. The relative expressions were calculated using the 2^−ΔΔCT^ method. The experiments were performed in triplicate and repeated three times.

### 2.6 Cell viability analysis

HCC cells were seeded in 96-well plates containing 0.2 mL medium for 24 h. After treatment with CDDP for 48 h, 20 μL of 3-(4,5-dimethylthiazol-2-yl)-2,5-diphenyltetrazolium bromide (MTT) was added into cell culture medium, followed by incubation at 37°C for 4 h. Dimethyl sulfoxide (150 μL per well) was added and incubated for 1 h. The absorbance was measured at 570 nm. The experiments were performed in triplicate and repeated three times.

### 2.7 Cell apoptosis analysis

Apoptosis rate of HCC cells in response to CDDP treatment was examined using Annexin V-fluorescein isothiocyanate (FITC) and PI (BD Biosciences, San Jose, CA, USA) according to the manufacturer’s instructions. Briefly, after treatments, cells (5 × 10^5^ cells/well in six-well plate) were harvested, washed, suspended in 100 µL binding buffer, and then stained with Annexin V-FITC and PI for 15 min in the dark at room temperature. Apoptotic cells were measured using the Cytoflex (Beckman Coulter, Inc.). FlowJo software was used to analyze the cytometric data. The experiments were performed in triplicate and repeated three times.

### 2.8 Clonogenic assay

After treatment with CDDP, parental or CDDP-resistant liver cancer cells were seeded onto six-well plates at a density of 5 × 10^4^ cells/well with normal or low glutamine medium. Cells were cultured for 2 weeks, and the medium was re-freshed every 3 days. The colonies were fixed by 4% paraformaldehyde and stained with 0.1% crystal violet for 5 min. Plates were washed extensively with phosphate-buffered saline (PBS). Colonies with >50 cells/colony were counted and recorded under a bright field microscopy. The experiments were repeated three times.

### 2.9 RNA immunoprecipitation (RIP)

Total RNA was isolated from HCC cells using an Rneasy Mini kit (Qiagen, Hilden, Germany) according to the manufacturer’s instruction. RIP assay was performed using a Magna RIPTM RNA-binding protein immunoprecipitation kit (Millipore, Bedford, MA, USA). Briefly, cells were lysed in RIP lysis buffer. Anti-IgG (control) or anti-PTBP1 (dilution 1:50) antibody with A/G immunomagnetic beads were added into cell lysates to immunoprecipitate PTBP1–RNA complexes. Purified RNA samples were subjected to qPCR analysis using GLS specific primers to determine the enrichment of GLS mRNA. The experiments were repeated three times.

### 2.10 RNA pull-down assay

The GLS mRNA-associated PTBP1 protein was examined by RNA pull-down assay. Briefly, negative control RNA (antisense RNA of binding motif) and the predicted binding motif of GLS were labeled using a biotin RNA labeling mix (Roche, Shanghai, China). The biotin labeled RNA was treated with RNase-free DNase I and purified by an RNeasy mini kit (Qiagen, Shanghai, China), followed by incubating with proteins from HCC cell. The streptavidin agarose beads (Invitrogen, Carlsbad, CA, USA) were then added into the mixture. After washing, the amount of PTBP1 protein in the RNA–protein complex was determined by western blot. The experiments were repeated three times.

### 2.11 RNA stability

The effect of PTBP1 on the stability of GLS mRNA was evaluated by detecting the half-life of GLS mRNA. Cells were treated with 5 μg/mL actinomycin D for 0, 2, 4, 6, and 8 h. Total RNA was collected by TRIzol reagent. The expression level of GLS mRNA was measured by qRT-PCR. The experiments were performed in triplicate and repeated three times.

### 2.12 Western blot

Liver cancer cells were harvested and washed at 4°C with PBS. Cells were lysed on ice with RIPA buffer (Thermo Fisher Scientific, Waltham, MA, USA) supplemented with 1× protease inhibitors cocktail (Thermo Fisher Scientific) for 20 min, followed by centrifugation at 12,000×*g* for 20 min at 4°C. Equal amount of protein sample (40 µL) from each group was separated by 10% SDS-PAGE and transferred onto polyvinylidene fluoride membranes. Membranes were blocked with 4% bovine serum albumin in phosphate-buffered saline with Tween 20 (PBST) for 1 h at room temperature, followed by incubation with primary antibodies at 1:1,000 dilution at 4°C for overnight. After complete washing by PBST, membranes were incubated with secondary antibodies at room temperature for 1 h. β-Actin was loading control. Membranes were detected by the enhanced chemiluminescence (Applygen Technologies Inc., Beijing, China) and the ChemiDoc Imaging Systems (Bio-Rad, Hercules, CA, USA). The experiments were repeated three times.

### 2.13 Statistical analysis

Statistical analysis was performed using the Prism software version 7.0 (GraphPad Software, Inc., La Jolla, CA, USA). Data were presented as mean ± standard deviation. Two-tailed student’s *t*-test was applied to analyze the statistical significance between two groups. Significance of difference among three or more groups was analyzed by one-way ANOVA. *p*-Value <0.05 was considered statistically significant.

## 3 Results

### 3.1 PTBP1 is upregulated in liver cancer and associated with CDDP resistance

**Figure 1:**
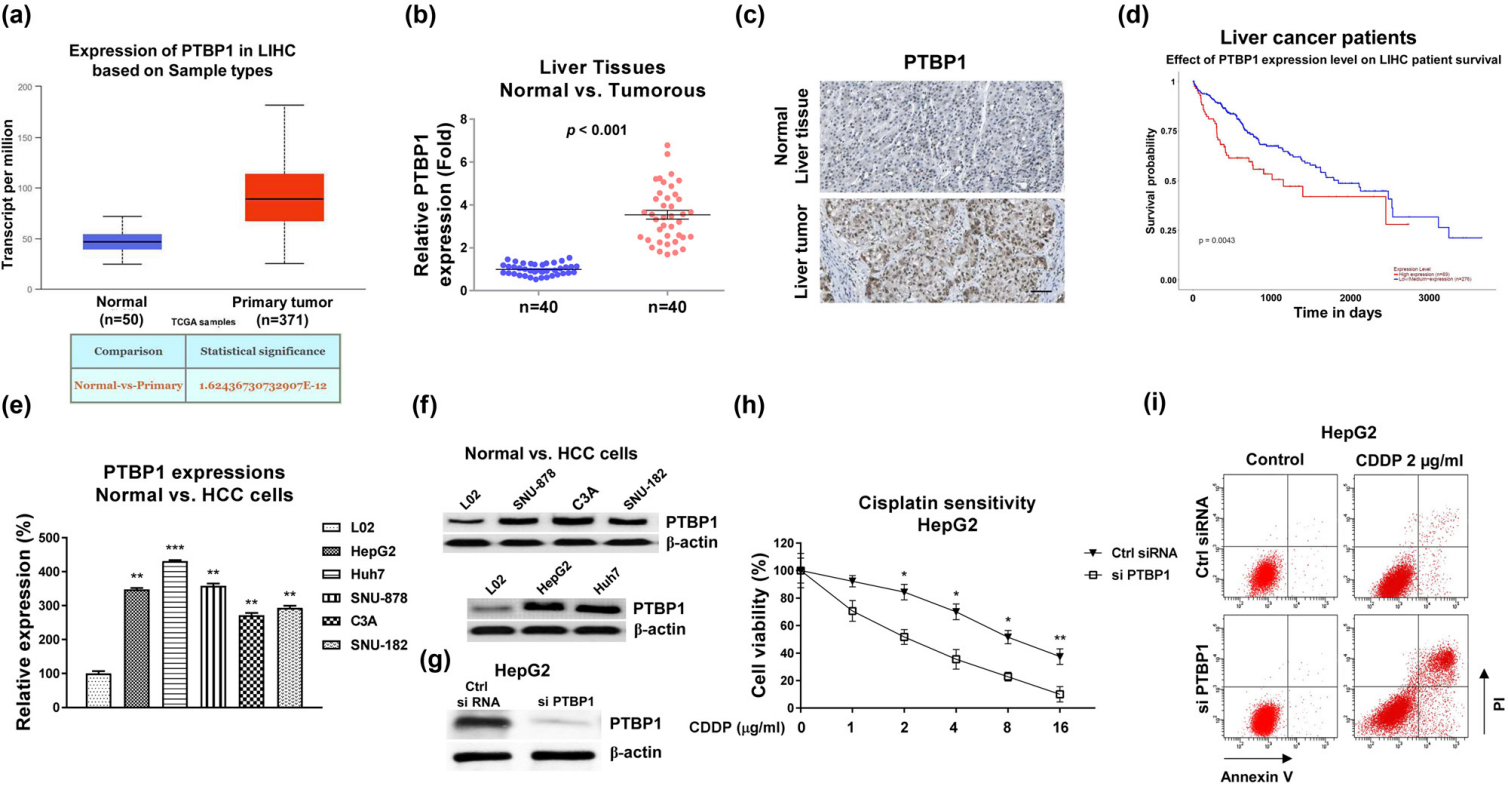
PTBP1 is positively correlated with liver cancer and CDDP resistance. (a) Expressions of PTBP1 in liver cancer tissues and normal liver tissues analyzed from TCGA database. (b) Expressions of PTBP1 in liver cancer tissues (*n* = 40) and normal liver tissues (*n* = 40) examined by qRT-PCR. (c) Representative IHC staining of PTBP1 in normal and tumorous liver tissues. (d) Kaplan–Meier plotter survival analysis shows the correlation between PTBP1 expression levels and survival rates of liver cancer cells. (e) mRNA and (f) protein expressions of PTBP1 are shown in one normal hepatocyte cell line and five HCC cell lines. (g) HepG2 cells were transfected with control siRNA or PTBP1 siRNA, expressions of PTBP1 were detected by western blot. (h) The above transfected cells were treated with CDDP at the indicated concentrations for 48 h. Cell response to CDDP was determined by cell viability assay and (i) Annexin V apoptosis assay. *p < 0.05; **p < 0.01; ***p < 0.001.

**Figure A1:**
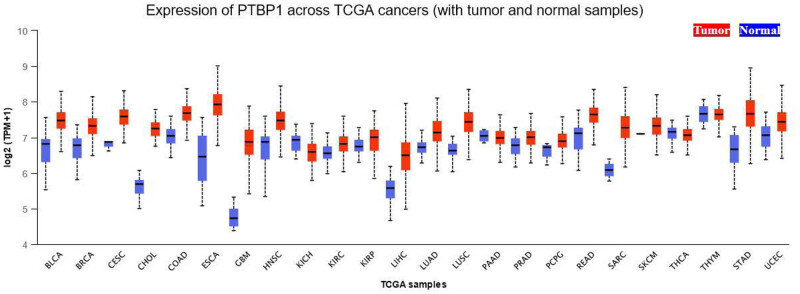
Expression of PTBP1 across TCGA cancers (normal vs tumor).

**Figure A2:**
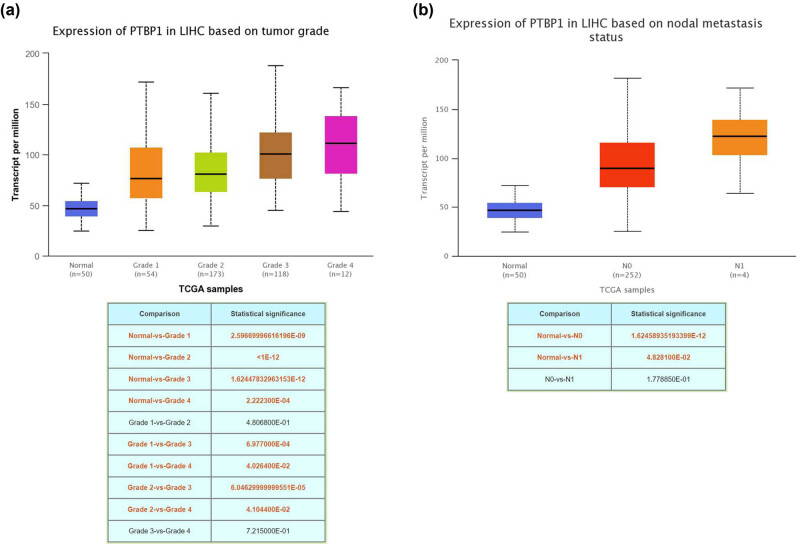
Expression of PTBP1 in HCC tissues based on (a) Tumor grad and (b) nodal metastasis status.

Bioinformatics analysis showed that PTBP1 was markedly overexpressed in various cancers, including liver cancer (Figure A1, Figure 1a). Moreover, the expression levels of PTBP1 were positively associated with the grades (Figure A2a) and metastasis status (Figure A2b) of liver cancer, suggesting a potentially oncogenic role of PTBP1 in liver cancer. To verify this, PTBP1 expressions were examined in human HCC specimens and their adjacent normal liver tissues from 40 HCC patients. qRT-PCR and immunohistochemistry showed that PTBP1 was remarkably highly expressed in cancerous tissues compared with normal tissues (Figure 1b and c). In addition, Kaplan–Meier plotter survival analysis illustrated that HCC patients with higher PTBP1 were significantly associated with lower survival rates (Figure 1d). Furthermore, both mRNA and protein levels of PTBP1 were significantly elevated in HCC cell lines, including HepG2, SNU-878, Huh7, C3A, and SNU-182 cells compared with normal hepatocytes cell line, L02 (Figure 1e and f). To explore the biological functions of PTBP1 in chemosensitivity, specific PTBP1 siRNA was transfected into HepG2 cells to knockdown PTBP1 expression (Figure 1g). Subsequently, HepG2 cells without or with PTBP1 silencing were exposed to increased concentrations of CDDP. Consistent results from cell viability assay and cell apoptosis assay demonstrated that silencing PTBP1 effectively enhanced the cytotoxicity of CDDP on HCC cells (Figure 1h and i). Taken together, these results suggest that PTBP1 is associated with poor response to CDDP treatment and exhibits an oncogenic role in HCC.

### 3.2 CDDP-resistant HCC cells display glutamine addictive phenotypes

**Figure 2:**
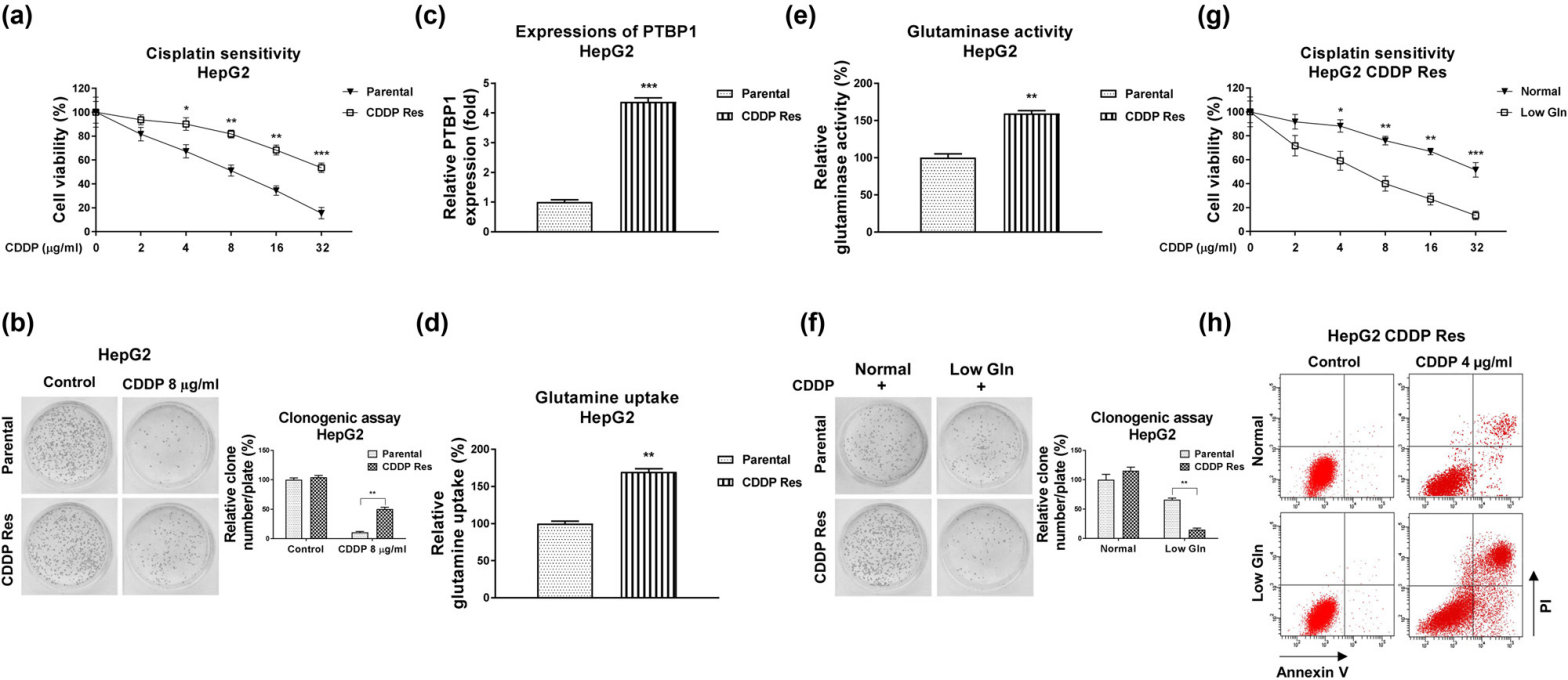
CDDP-resistant liver cancer cells show glutamine addictive phenotypes. (a) HepG2 parental cells and CDDP-resistant cells were treated with CDDP at the indicated concentrations for 48 h. Cell response to CDDP was determined by cell viability assay and (b) clonogenic assay. (c) Expressions of PTBP1 in HepG2 parental cells and CDDP-resistant cells were examined by Western blot. (d) Glutamine uptake and (e) GLS activity were examined in HepG2 parental and CDDP-resistant cells. (f) HepG2 parental cells and CDDP-resistant cells were cultured with normal or low glutamine condition, cells were treated with CDDP at the indicated concentrations. The cell survival rates were examined by clonogenic assay, (g) cell viability assay, and (h) Annexin V apoptosis assay. *p < 0.05; **p < 0.01; ***p < 0.001.

**Figure A3:**
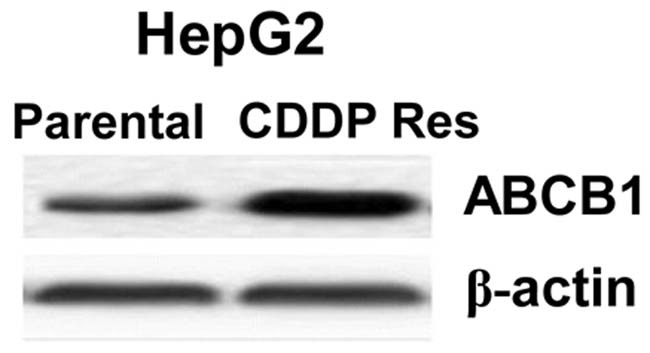
Expressions of ABCB1 in parental and CDDP resistant HepG2 cells.

We then investigated the underlying cellular mechanisms of the PTBP1-promoted CDDP resistance. Accumulating evidence unveiled that the dysregulated cancer metabolism was tightly associated with tumorigenesis, progressions as well as chemoresistance [9,10]. To assess the roles of glutamine metabolism in CDDP sensitivity, we established a CDDP-resistant liver cancer cell line (HepG2 CDDP Res) via exposing cells to elevated concentrations of CDDP. HepG2 parental cells were maintained in cell culture medium and treated with CDDP (4–16 μg/mL) for at least 4 weeks to select the survival (CDDP Res) cells. As shown in Figure 2a, HepG2 CDDP Res cells could tolerate higher concentrations of CDDP than HepG2 parental cells (Figure 2a and b). The CDDP IC50 of HepG2 CDDP Res was 35.24 µg/mL, which is significantly higher than that of HepG2 parental cells (8.24 µg/mL) (Figure 2a). Consistent results from clonogenic assay demonstrated that CDDP treatment at 8 µg/mL only slightly inhibited cell survival of HepG2 CDDP-resistant cells. However, apparently cell death was observed in HepG2 parental cells under the same concentration of CDDP treatment (Figure 2b). Moreover, the ABCB1 protein expression was significantly upregulated in CDDP-resistant HepG2 cells (Figure A3). Expectedly, PTBP1 was significantly upregulated in HepG2 CDDP-resistant cells compared to that in HepG2 parental cells (Figure 2c). We then evaluated the glutamine metabolism characteristics in CDDP-resistant liver cancer cells. The glutamine uptake (Figure 2d) and GLS activity (Figure 2e), two glutamine metabolism readouts were significantly elevated in HepG2 CDDP-resistant cells. Furthermore, under low glutamine supply, CDDP-resistant cells were more inhibited by CDDP treatment, while the HepG2 parental cells were less affected under the same CDDP treatment (Figure 2f). Subsequently, MTT assay (Figure 2g) and cell apoptosis assay (Figure 2h) demonstrated that low glutamine treatment effectively re-sensitized CDDP-resistant cells to CDDP treatment. Summarily, these results revealed that CDDP-resistant HCC cells were more dependent on glutamine metabolism, suggesting that targeting glutamine metabolism is a potentially therapeutic approach for treatment of chemoresistant liver cancer.

### 3.3 PTBP1 promotes glutamine metabolism of HCC cells

**Figure 3:**
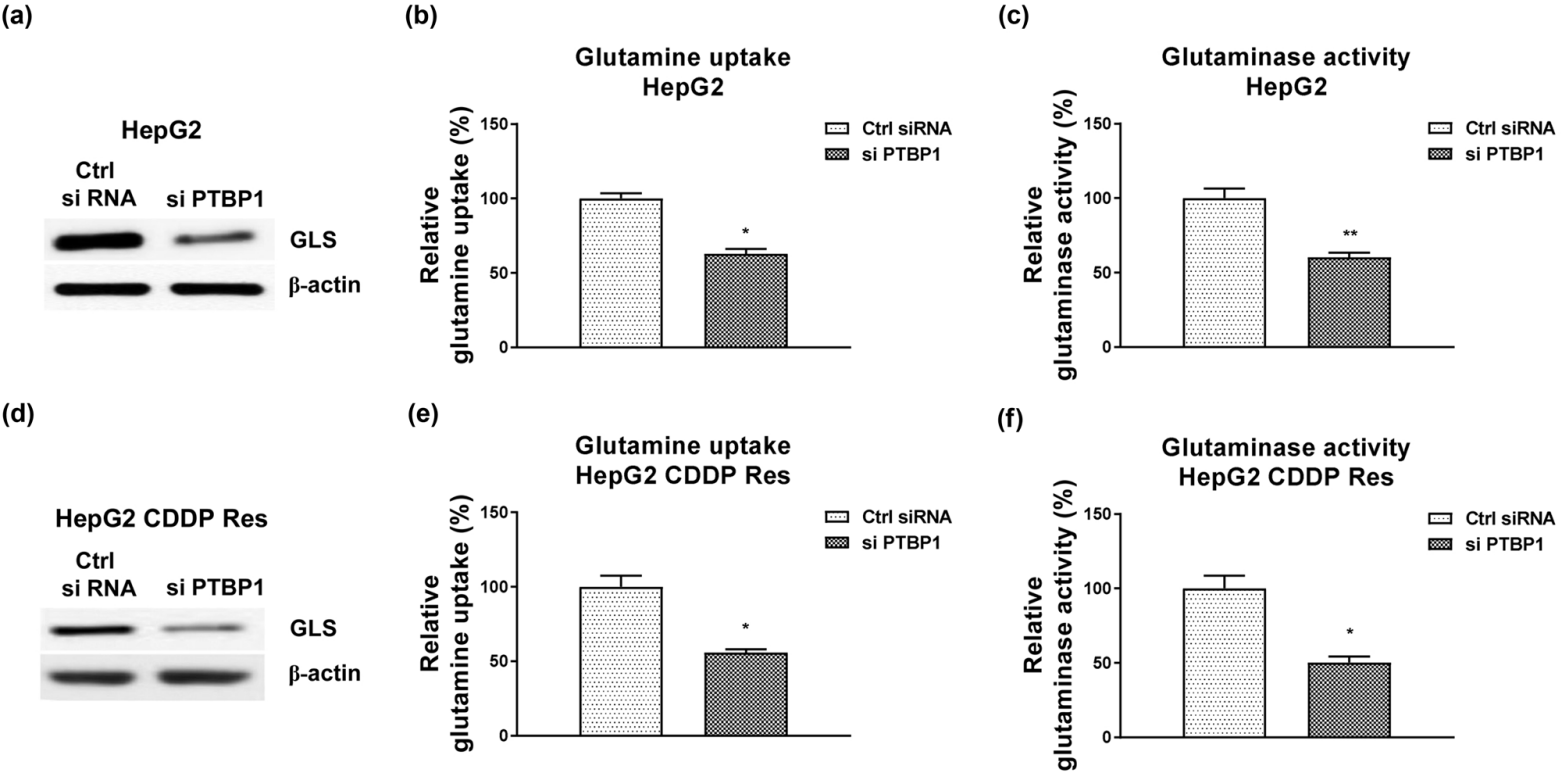
PTBP1 promotes glutamine metabolism of HCC cells. (a) HepG2 cells were transfected with control siRNA or siPTBP1. Expressions of GLS were examined by western blot. (b) Glutamine uptake and (c) GLS activity from the above transfected cells were examined. (d) HepG2 CDDP Res cells were transfected with control siRNA or siPTBP1. Expressions of GLS were examined by western blot. (e) Glutamine uptake and (f) GLS activity from the above transfected cells were examined. *p < 0.05; **p < 0.01.

**Figure A4:**
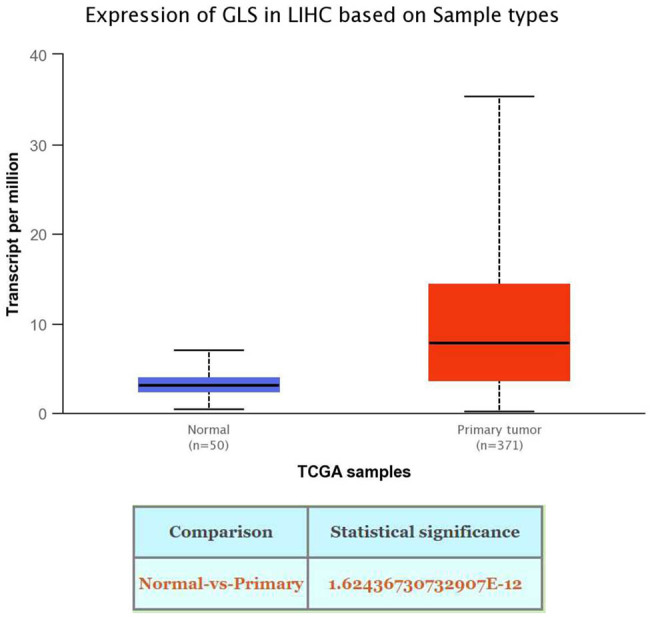
Expression of GLS in normal liver tissue and HCC tissues from TCGA database.

Given the above results revealed a PTBP1-promoted CDDP resistance and a positive correlation between glutamine metabolism and CDDP resistance in HCC cells, we then evaluated whether PTBP1 directly regulated glutamine metabolism of HCC cells. As shown in Figure 3a, knocking down of PTBP1 significantly blocked the protein expression of GLS, which catalyzes the speed-limiting reaction of glutamine metabolism in HCC cells and was significantly upregulated in liver cancer (Figure A4). Consistently, silencing PTBP1 effectively suppressed glutamine uptake (Figure 3b) and GLS activity of HCC cells (Figure 3c). We then assessed the effects of PTBP1 knockdown on glutamine metabolism of CDDP-resistant HCC cells. Expected results demonstrated that knocking down of PTBP1 significantly downregulated the GLS expression (Figure 3d) and suppressed glutamine metabolism (Figure 3e and f) of HepG2 CDDP Res cells. In summary, these results indicated that PTBP1 positively regulates glutamine metabolism of liver cancer cells.

### 3.4 PTBP1 binds to 3′-UTR of GLS to upregulate GLS expressions via preventing its RNA degradation

**Figure 4:**
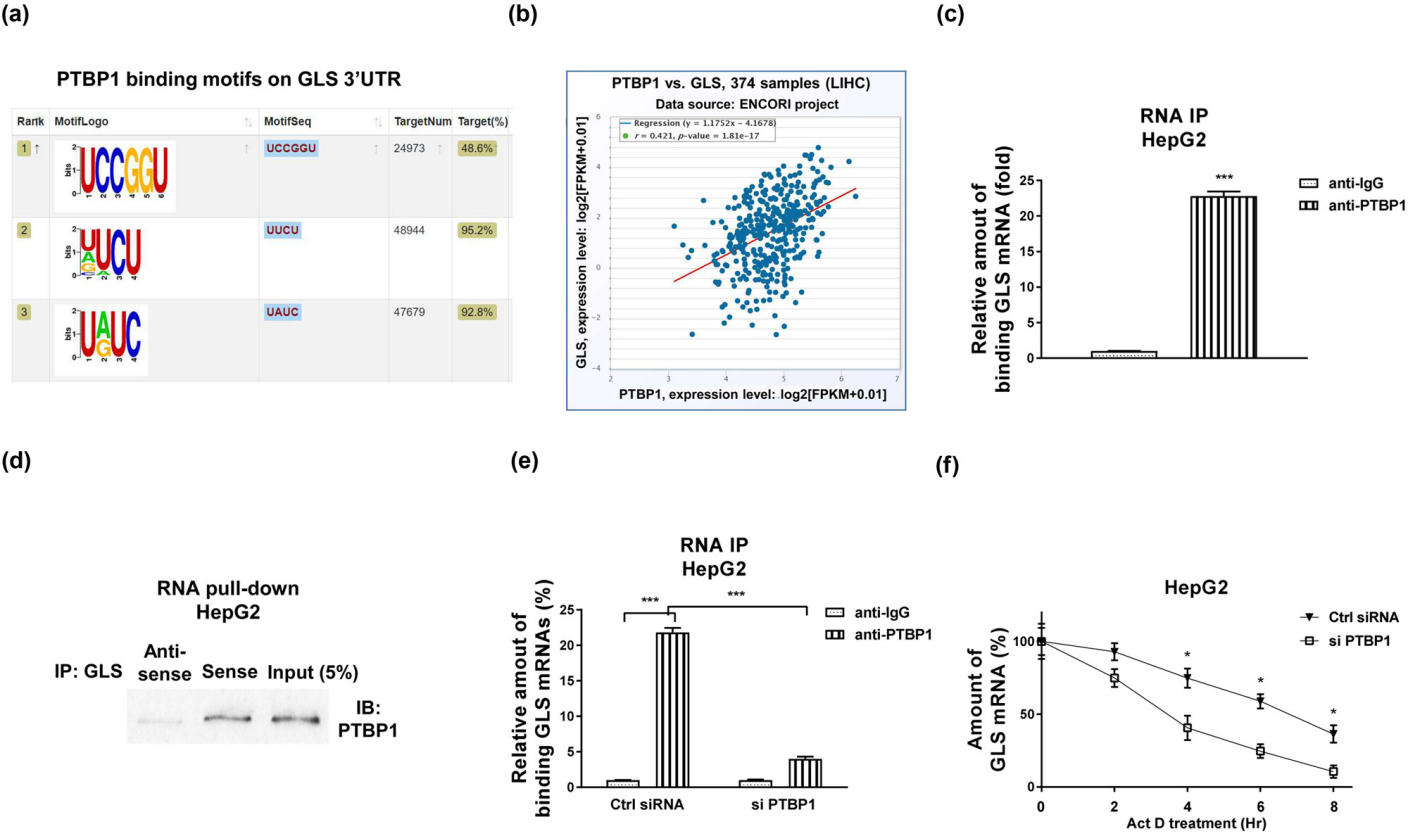
PTBP1 binding to 3′-UTR of GLS mRNA to regulate its mRNA stability. (a) Predicted binding motifs of PTBP1 on GLS 3′-UTR. (b) Expression correlation analysis between GLS and PTBP1 in liver cancers. (c) RIP was performed in HepG2 cells using IgG control or anti-PTBP1 antibody. GLS mRNA amounts in the PTBP1-immunoprecipitated fraction were measured by qRT-PCR. (d) RNA pull-down assay was performed in HepG2 cells. The PTBP1 protein which was associated with GLS mRNA was assayed by western blotting. (e) HepG2 cells were transfected with control siRNA or PTBP1 siRNA for 48 h, RIP experiments were performed and the GLS mRNA abundance in immunoprecipitated fraction was determined by qRT-PCR. (f) HepG2 cells were treated with 5 μg/mL actinomycin D for 0, 2, 4, 6, and 8 h, the relative half-life of GLS mRNA levels were measured by qRT-PCR. *p < 0.05; ***p < 0.001.

**Figure A5:**
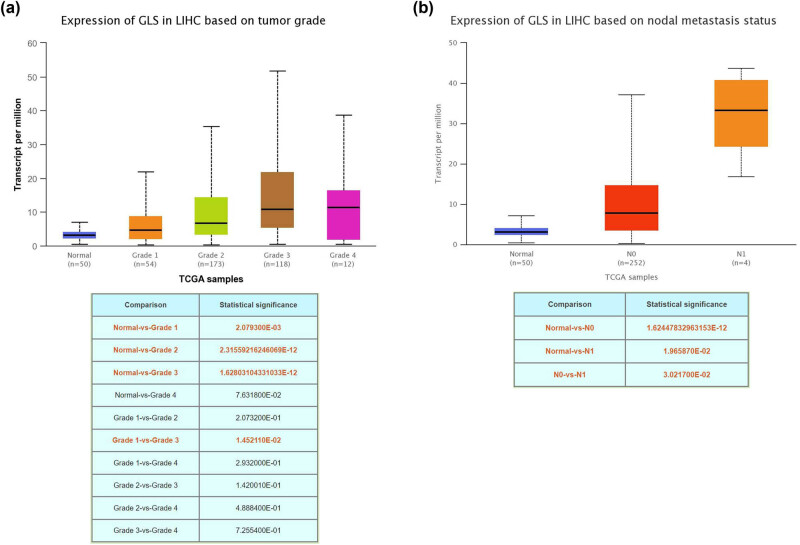
Expression of GLS in HCC tissues based on (a) Tumor grad and (b) nodal metastasis status.

Recent studies revealed that PTBP1 acted as a typical RNA-binding protein [12]. Thus, the post-transcriptional regulation of target genes such as regulating RNA stability might be a potential molecular mechanism of the PTBP1-promoted glutamine metabolism. We then analyzed the downstream RNA targets of PTBP1. Analysis from TCGA database indicated that GLS was significantly upregulated in liver cancer (Figure A4) and positively associated with the grades (Figure A5A) and metastasis status (Figure A5b) of liver cancer. Previous studies indicated that PTBP1 preferentially bond to polypyrimidine-rich stretches of RNAs [12,19]. We then assessed whether GLS mRNA contains PTBP1 binding elements. Bioinformatics analysis illustrated a PTBP1–RNA interaction between PTBP1 and GLS 3′-UTR which contains multiple PTBP1 binding motifs (Figure 4a). Among them, one motif showed the strongest binding capacity with PTBP1 (Figure 4a). Expectedly, a significantly positive correlation between PTBP1 and GLS was observed in HCC patients (Figure 4b), suggesting that PTBP1 positively regulates GLS mRNA through binding to its 3′-UTR region. We then hypothesized PTBP1-enhanced mRNA stability of GLS to upregulate its expression. To test that, RIP assay was performed using PTBP1-specific antibody in HepG2 cells. qRT-PCR results demonstrated that GLS 3′-UTR region was enriched in PTBP1-precipitated RNA fragments (Figure 4c). In addition, RNA pull-down assay was performed using biotin-labeled GLS 3′-UTR to pull down specific binding proteins. Results in Figure 4d showed that significant amount of PTBP1 was pulled down by GLS 3′-UTR. To examine whether the PTBP1-upregulated GLS expressions was through binding to 3′-UTR of GLS, PTBP1 was silenced in HepG2 cells and the PTBP1–GLS interaction was analyzed by RIP. Expectedly, HepG2 cells with lower PTBP1 bond less amount of mRNA fragments (Figure 4e). We then evaluated whether PTBP1 affects mRNA stability of GLS. RNA stability assays were performed to compare the half-life of GLS mRNAs in control and PTBP1-silenced HCC cells. Results showed that the half-life of GLS mRNA was significantly suppressed in PTBP1-silencing cells compared to that in control cells (Figure 4f). Taken together, these results validated that PTBP1 upregulated GLS expression through binding to GLS 3′-UTR, resulting in GLS mRNA stabilization in HCC cells.

### 3.5 Blocking PTBP1 re-sensitizes CDDP-resistant HCC cells through suppressing glutamine metabolism

**Figure 5:**
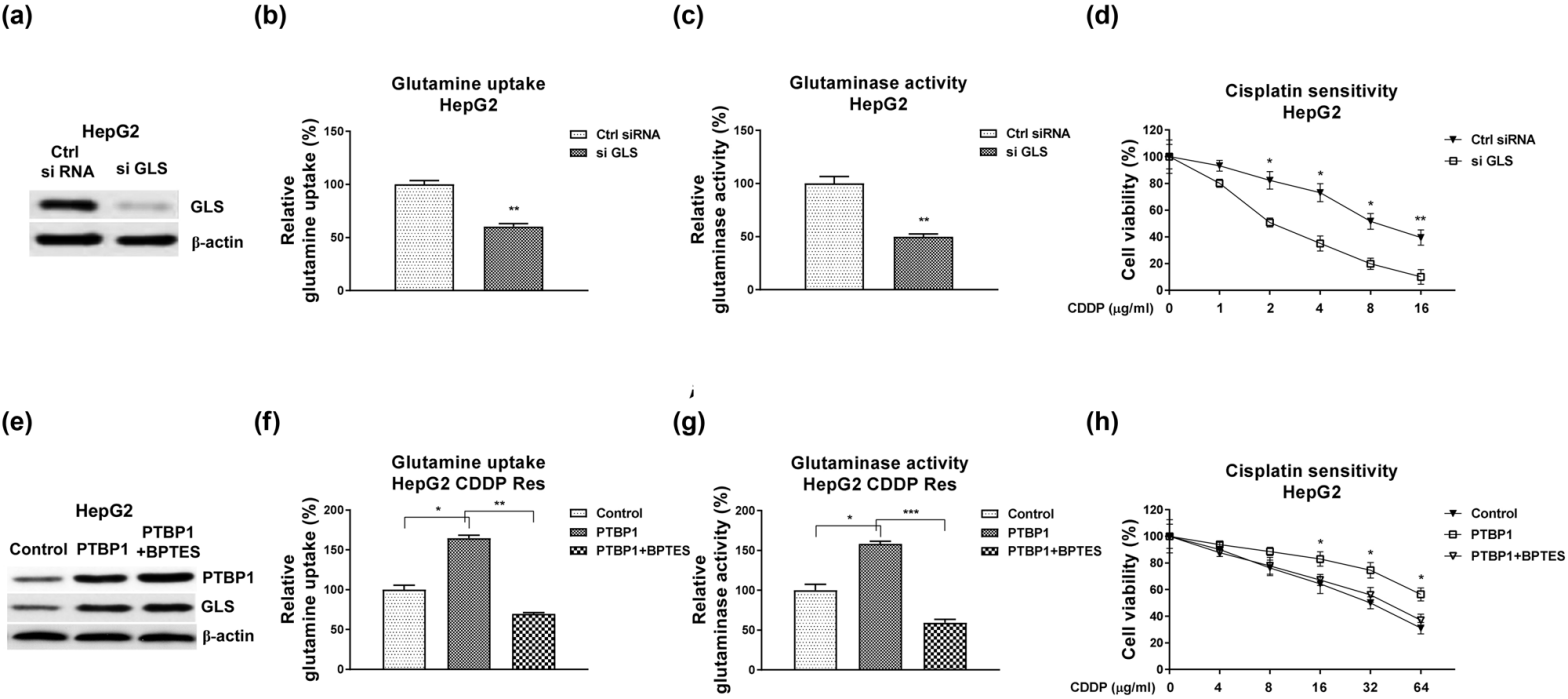
Roles of the PTBP1–GLS–glutamine metabolism pathway in CDDP-resistant HCC cells. (a) HepG2 CDDP-resistant cells were transfected with control siRNA or si GLS, expressions of GLS were determined by western blot. (b) Glutamine uptake, (c) GLS activity, and (d) CDDP sensitivity were examined in the above transfected cells. (e) HepG2 CDDP-resistant cells were transfected with control plasmid or GLS overexpression plasmid. Cells were treated with control or GLS inhibitor, BPTES. Expressions of GLS were determined by western blot. (f) Glutamine uptake, (g) GLS activity, and (h) CDDP sensitivity were examined in the above transfected cells. *p < 0.05; **p < 0.01; ***p < 0.001.

Finally, we assessed whether the PTBP1-promoted CDDP resistance was through upregulating the GLS-mediated glutamine metabolism. Silencing GLS (Figure 5a) effectively blocked glutamine uptake (Figure 5b), GLS activity (Figure 5c), and the CDDP resistance of HepG2 (Figure 5d). Moreover, HepG2 CDDP Res cells with PTBP1 overexpression (Figure 5e) displayed significantly elevated glutamine uptake (Figure 5f), GLS activity (Figure 5g), and the CDDP resistance (Figure 5h). Consequently, these phenotypes were further overridden by treatment of GLS inhibitor, BPTES, which is a noncompetitive inhibitor of GLS to specifically block the GLS enzymatic activity but not affect the protein expression (Figure 5e–g). Taken together, the above results demonstrated a PTBP1–GLS–glutamine metabolism axis in regulating CDDP resistance of liver cancer cell.

## 4 Discussion

Liver cancer is a prevalent malignancy with a high mortality rate and poor prognosis throughout the world. HCC is the most common liver cancer subtype [1–3]. Currently, the platinum-based anti-cancer drugs have been widely applied to improve the clinical outcomes of liver cancer patients who were diagnosed at middle or advanced stage [5,6]. However, development of drug resistance impaired the clinical applications of CDDP. This study unveiled a PTBP1-promoted CDDP resistance in HCC cells through modulating glutamine metabolism. PTBP1 was significantly upregulated in liver tumors and cell lines. Moreover, PTBP1 was positively associated with CDDP resistance, indicating that PTBP1 is a potentially diagnostic biomarker and therapeutic target against chemoresistant liver cancer.

Cancer cells display a new hallmark that they reprogram glucose metabolism by utilization of glucose toward aerobic glycolysis instead of mitochondrial oxidative phosphorylation, a phenomenon called “Warburg effect” [20]. Not surprisingly, cancer cells demand a variety of nutrients such as glucose, glutamine, and lipids as carbon sources and energy for hyper-proliferation [20]. Since oncogenic shift of cellular metabolism rendered cancer cells addicted to glutamine, molecular targets and signaling pathway involved in glutamine metabolism could be potentially therapeutic approaches. Therefore, in light of the glutamine metabolism in the CDDP-resistant HCC cells, investigating new therapeutic targets and underlying molecular mechanisms are urgent tasks to overcome CDDP resistance. From the established CDDP-resistant liver cancer cell line, we observed that the glutamine metabolism was remarkably elevated. Moreover, under low glutamine supply, CDDP-resistant HepG2 cells exhibited higher CDDP sensitivity than that from HepG2 parental cells. We further demonstrated that PTBP1 stimulated glutamine metabolism of HCC cells. These results consistently revealed that PTBP1 promoted CDDP resistance of HCC cells through modulating the glutamine metabolism.

As an RNA binding protein, PTBP1 preferentially binds to polypyrimidine-rich stretches of RNA to regulate various RNA processions such as RNA metabolism, alternative splicing, translation, stability, translocation, and pre-mRNA processing [12]. In addition, PTBP1 was an important regulator in multiple cancers [13–16]. However, the roles of PTBP1 in glutamine metabolism as well as the direct mechanisms of GLS regulation have not been investigated. Here, we highlighted a PTBP1 binding motif in 3′-UTR of GLS. The binding of PTBP1 on GLS 3′-UTR was validated by RIP assay and RNA pull-down assay. Previous studies reported that PTBP1 bond to 3′-UTR of downstream mRNAs to stabilize them by preventing the degradation protein UPF1 from binding to 3′-UTRs [21]. Results from RNA stability assay consistently showed that PTBP1 bond to GLS 3′-UTR to stabilize its mRNA. These results consolidated that PTBP1 upregulated GLS expressions through direct binding to 3′-UTR of GLS, leading to the stabilization of GLS mRNA. However, this study still has limitations that the majority of discovery was from *in vitro* assay. An *in vivo* xenograft mouse model will be analyzed in our further works.

In summary, this study unveiled a PTBP1-promoted CDDP resistance in HCC cells through promoting glutamine metabolism by stabilizing the GLS mRNA. Our discovery highlights a novel molecular axis for overcoming chemoresistant liver cancer.
